# Indoor Air Quality Real-Time Monitoring in Airport Terminal Areas: An Opportunity for Sustainable Management of Micro-Climatic Parameters

**DOI:** 10.3390/s18113798

**Published:** 2018-11-06

**Authors:** Sara Zanni, Francesco Lalli, Eleonora Foschi, Alessandra Bonoli, Luca Mantecchini

**Affiliations:** 1 Department of Civil, Chemical, Environmental and Materials Engineering—DICAM, University of Bologna, via Terracini 28, 40131 Bologna, Italy; sara.zanni7@unibo.it (S.Z.); francesco.lalli2@unibo.it (F.L.); eleonora.foschi3@unibo.it (E.F.); alessandra.bonoli@unibo.it (A.B.); 2 Department of Civil, Chemical, Environmental and Materials Engineering—DICAM, University of Bologna, viale del Risorgimento 2, 40136 Bologna, Italy

**Keywords:** indoor air quality, airport terminal, micro-climatic management

## Abstract

Indoor air quality (IAQ) management in public spaces is assuming a remarkable importance. Busy environments, like airport terminals, are currently regarded as possible hotspots and IAQ is a crucial element for passengers and staff protection, as well as a key aspect of airport passenger experience. A one-month monitoring period has been performed on IAQ in the airport of Bologna (Italy), as prototypal example of large regional airport. Four strategic areas within the airport have been equipped with electronic monitoring platforms, including different contaminants and two microclimatic sensors. Data suggest that daily variation in IAQ parameters typically follow the activity pattern of the different environments under study (i.e., passengers’ flows) for gaseous contaminants, where particulate matter counts oscillate in a definite range, with a significant role played by ventilation system. Gaseous contaminants show a correlation between indoor and outdoor concentrations, mainly due to airside activities. Micro-climatic comfort parameters have been tested to match with standards for commercial environments. As results appears in line with typical households IAQ values, the current air ventilation system appears to be adequate. Nevertheless, an integrated air management system, based on real-time monitoring, would lead to optimization and improvement in environmental and economical sustainability.

## 1. Introduction

Air transport plays a crucial role in global economic development and in the last decade air traffic has increased dramatically worldwide, with a significant growth in atmospheric emissions. 

During the last forty years, the worldwide aviation industry has increased, on average, by 5% yearly. At the same time, emissions from civil aviation activities have increased accordingly and, in particular, greenhouse gas emissions from international aviation have grown by 87% since 1990 [1]. According to recent studies and forecasts [2,3,4,5], the annual rate of air traffic growth should be around 4–5% in the next years: this expected further growth of the sector and the parallel development of city airports—often included in urban areas—have increased the attention on the environmental effects that growing airport traffic could generate on the communities living around airports and, at the same time, have put even growing pressures on governments and regulators to improve the development of effective environmental policies.

The impacts produced by airport activities—from the aircraft Landing and Take-Off cycle (LTO) to handling operations and ground support equipment, concerning air-side activities, and to airport ground access systems to passenger, baggage, and freight terminal operations, concerning land-side activities—have received increasing attention during the last years [6,7,8,9,10,11]. In the airport surroundings, monitoring systems provide data used to build and manage models able to identify the contribution of airport activities to outdoor pollutant concentration, with special regard to nitrogen oxides (NO_x_), volatile organic compounds (VOCs), hydrocarbons (HC), particulate matter (PM), and carbon monoxide (CO) [12,13,14,15,16]. Evidences from the literature has shown that airport operations significantly contribute to annual pollutants concentration at the airport boundaries. In particular, CO concentrations in the vicinity of the terminals were found to be highly dependent on aircraft movement, whereas NO_x_ concentrations were dominated by emissions from ground support vehicles [17,18,19].

Service quality and passenger satisfaction are topics of primary interest in the airport industry. Direct and indirect surveys campaigns have been systematically carried out by international agencies (Airport Council International and IATA), as well as frequent ad hoc initiatives by single airports [20]. Due to increasing traffic and changes in the air transport market, it has become more important for airport operators to monitor and analyze relevant data and information regarding passengers’ perception on Airport Service Quality (ASQ), by complying national and international rules. Even if the reduction of any negative environmental impacts is crucial for airport management, it is worthless if the airports are not healthy and comfortable places to live and work in. Indoor environmental quality, including indoor thermal, lighting, acoustic, and air quality, is of paramount importance for the well-being of travelers and airport workers. Therefore, the progressive evolution towards sustainable and low carbon airport terminal buildings should not be successfully performed without tackling these aspects.

Airport terminal buildings are composed by areas accomplishing different functions, such as the departure lounge with boarding gates and shopping areas, security control areas, check-in area, baggage claim hall, arrival hall, etc. Different zones have different heating and cooling demand, due to different occupant density, activity performed, or time spent by travelers. These issues make the management of indoor environmental conditions challenging. Moreover, the passenger flow in airport terminals varies significantly throughout the year or even throughout a single day, due to the flight schedule, leading to a worsening of the terminal indoor environment quality.

Indoor air quality (IAQ) assessment becomes, consequently, a key element for the environmental quality evaluation of airport terminals, but it is necessary to identify technological solutions suitable for the purpose. Several reviews of opportunities currently offered in the field are available [21,22,23] presenting analytical capabilities of the different monitoring methods, with particular regards to accuracy, precision, threshold levels for detection, time resolution, comparability, and data completeness. 

The fluctuations in the level of activity and crowding, typical of terminal environment, would more likely be described by the application of a continuous monitoring method, able to identify possible repetitive patterns of contamination and, therefore, support best management options. This confirms the widely recognized need for a broader application of monitoring on short time intervals (i.e., continuous or semi-continuous) [24] to build consistent and statistically relevant database, even if spot monitoring would offer higher precision and more information on composition, growth, and characteristics of indoor air pollutants (e.g., particle mass spectrometers).

In the authors’ opinion, the literature concerning airport indoor air quality appears scarce and not exhaustive and it is mostly linked to energy efficiency. Balaras et al. [25] investigated the energy consumption and the indoor environment quality of Hellenic airport terminal buildings in 2003 by means of direct surveys. The results show that almost 70% of the surveyed people complained about the poor indoor air quality, and remarkable differences were highlighted at various areas of the terminal. Numerical simulations performed by Meng et al. [26] show that by enhancing vertical ventilation to discharge hot air near the ceiling, the average number of dissatisfied passengers can be reduced and the energy consumption for heating and cooling can be saved. Finally, an interesting work of Wang et al. [27] shows that thermal environment and air quality are the two crucial factors for the overall satisfaction level, and travelers’ air quality satisfactory level is highly correlated with the CO_2_ concentration.

Within the presented framework, a two-month period of IAQ and micro-climatic parameter characterization has been performed in different areas of a medium-size airport terminal with the aim of identifying possible criticality and opportunity to improve environmental management.

## 2. Materials and Methods

The goal of the study was identified, in accordance with the test-site “Aeroporto Guglielmo Marconi di Bologna”, Italy, in performing a preliminary study of air quality in potentially critical airport areas. In particular, the efforts have been aimed at identifying airborne contamination patterns and their possible relationship with the characteristic activities of the specific environment (i.e., passengers’ flow, boarding operations, baggage reclaim). Bologna Airport is a medium-size airport (a large regional, following the EU classification), very close to the city center of Bologna, in Northern Italy. In 2017 approximately 72,000 aircraft movements and 8.2 million passengers were registered. 

**Figure 1:**
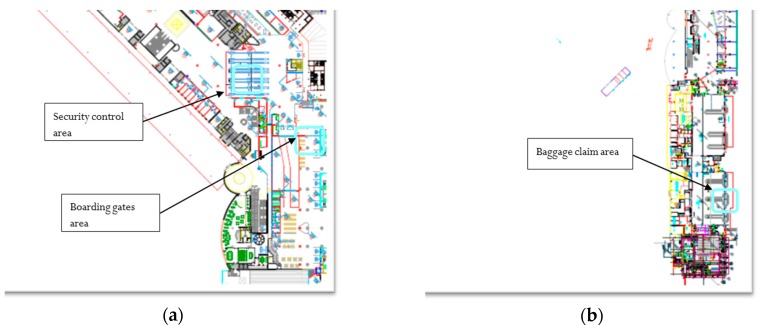
Terminal areas selected for monitoring phase: (**a**) security check lines and boarding gates (1st floor); and (**b**) baggage claim area (ground floor).

From the operative point of view, the study aimed to assess the effectiveness of the currently used ventilation and air treatment systems, both for IAQ and micro-climatic comfort management. The ventilation system currently applied is set for continuous functioning, 24/7, without any exchange rate adjustment. The experiment has been structured into two main phases of IAQ monitoring in different areas of the airport (see Figure 1), regarded as potentially critical for IAQ management, i.e., security check, waiting area for boarding and baggage claim (both landside and airside).

A monitoring technology has been applied at pilot scale, in order to investigate the IAQ and evaluate the opportunity for the scaling-up of the implementation to a full-time monitoring network. The monitoring platform is manufactured by Ideas & Motion S.r.l., v. Moglia 19, 12062 Cherasco (CN), Italy, while data management and visualization software is developed by Microsys s.r.l., v. Antonio da Recanate 1, 20124 Milano (MI), Italy. 

The electronic platform offers four slots for contaminants concentration sensors, together with two microclimatic sensors, for temperature and humidity. In particular, sensors equipped on each platform are:

Environmental parameters:

- Temperature (°C): [−40 °C to 125 °C], ±2 °C worse accuracy;
- Humidity (%RH): [0–100% RH], ±4%RH accuracy, referred to sensor’s reading;
- Air contaminants/odorous gases (Figaro TGS2602, by Figaro Engineering Inc., 1-5-11 Senbanishi, Mino, Osaka 562-8505, Japan) (ppm): [1 to 30 ppm], C_6_H_5_CH_3_ (toluene), H_2_S (hydrogen sulphide), CH_3_CH_2_OH (ethanol), NH_3_ (ammonia), H_2_ (hydrogen);
- Solvent gases/VOC (Figaro TGS2620, by Figaro Engineering Inc., 1-5-11 Senbanishi, Mino, Osaka 562-8505, Japan) (ppm): [50 to 5000 ppm], CH_3_CH_2_OH (ethanol), H_2_ (hydrogen), C_4_H_10_ (isobutene), CO (carbon monoxide), CH_4_ (methane);
- Particulate matter (Shinyei PPD42NS, by SHINYEI Technology Co., Ltd., 77-1 Kyomachi, Chuo-ku, Kobe 651-0178, Japan) (pcs/L):
  ○ PM_1_ (Shinyei PPD42NS): [0 to 28,000 pcs/L], detectable particle size 1–10 µm;
  ○ PM_2.5_ (Shinyei PPD42NS): [0 to 280,000 pcs/L], detectable particle size 2.5–10 µm.

One of the two monitoring platform is also equipped with two sensors (MiCS-4514, by SGX Sensortech, Corcelles-Cormondreche, Switzerland) for gaseous contaminants typically related to outdoor environment and possibly linked with the airside activity of an airport, i.e., CO and NO_2_. Both the airborne contaminants are related to combustion processes, but, where the former is generated in the presence of incomplete combustion of hydrocarbon-based fuel at low reaction temperature (i.e., in winter conditions, contributing to the traditional “smog”), the latter is the result of combustion in air (where nitrogen and oxygen are the most abundant gases) at high temperature and it is involved in the photochemical smog formation. For both contaminants, traffic is typically regarded as a major source [28,29].

Semiconductor sensors, such as the Figaro TGS2602 and Figaro TGS2620, consist of two elements: the first one, made of tin dioxide (SnO_2_) with crystalline structure, and the second one acting as a heater. The first one presents an excess of electrons due to the charging effect triggered by the second unit, making it sensitive to different gases concentrations. Once electrically supplied, resistance detected at the sensor (precision of ±1% in the whole range) is proportional to gases concentration. Through software conversion, gas concentration is displayed as parts per million (ppm) on the web platform. Since sensors are non-selective, i.e., they are sensible to groups of gases, not to single ones, they can be calibrated both with single and mixture of gases (ethanol, hydrogen, control mixture UNI EN 12619:2002, i.e., methane 2.0 mg/m^3^, ethane 1.5 mg/m^3^, toluene 0.5 mg/m^3^, benzene 0.5 mg/m^3^, methylene chloride 0.5 mg/m^3^, with oxygen 11%, carbon dioxide 10%, carbon monoxide 50 mg/m^3^, and nitrogen as complementary). The calibration performed and cross-referenced against a single device calibrated through standard method returns a final accuracy of ±15%, including resistance detection and calibration errors.

**Figure 2:**
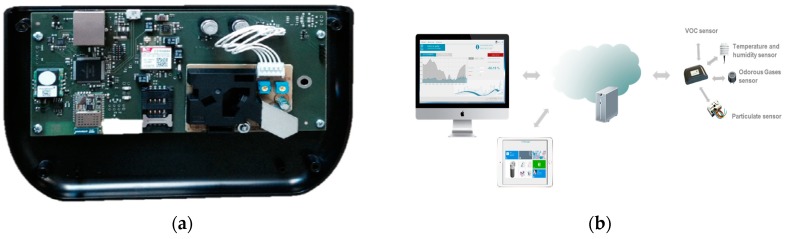
Monitoring platform detail: wiring board and sensors (**a**); and wireless communication system (**b**) (courtesy of U-Earth Biotechnologies)

The Shinyei PPD42NS laser scatter sensor detects particulate matter (PM), classifying them by size ranges, thanks to light beam alteration (Figure 2). A 100 Ω resistor provides a thermal plume, allowing particles inside the detection chamber, without ventilation. Through the projection of an infrared light beam, the sensor indirectly estimates the number of PM suspended in air by the scattering of the beam itself hitting particles traveling across the chamber. Photons are scattered with an approximate 45° angles, focused by a lens into a specific region where, finally, they are detected by a photodiode, translating light into a pulse signal, which is proportional to particles concentration. In this way, the measured parameter is the air opacity (opacity percentage or low pulse occupancy), defined as the percentage of time (relative to a predefined time interval, e.g., 1 s) in which particles are detected by the photo diode sensor. The particle detection threshold of the sensor is determined by a pass-band filter, removing part of the background noise and identifying the range of particles counted. In particular, the two channels allow two size ranges count (i.e., 1–10 microns and 2.5–10 microns) with accuracy related to PM concentration (±1.5% for concentrations below 5%; ±2.5% for concentrations above 15%, i.e., the sensor’s saturation limit), with no indication whether could be related to difference in the total mass, size distribution, or optical properties of the lens, or a combination of these phenomena.

Sensors may, therefore, present quite a remarkable drawback, due to relatively low accuracy and reading method (e.g., for particulate sensor), but it can be overcome in two ways:

1. statistical analysis of the broad population of data collected (one sample every five minutes per sensor, makes 288 concentration data per each sensor per day);
2. evaluation of trends and possible repetitive patterns in contamination: rather than treating data as absolute values, they are elaborated in comparison to a baseline (in the present study, in percentage increase over the minimum value detected). Each sensor collects data every five minutes, but it can be set to detect concentration and environmental data on five minutes to one-hour basis. Thanks to wireless sensor network (WSN) technology, it can communicate data to a server via Wi-Fi, GSM network or Ethernet, every determined interval, making them available almost in real-time for displaying.

**Figure 3:**
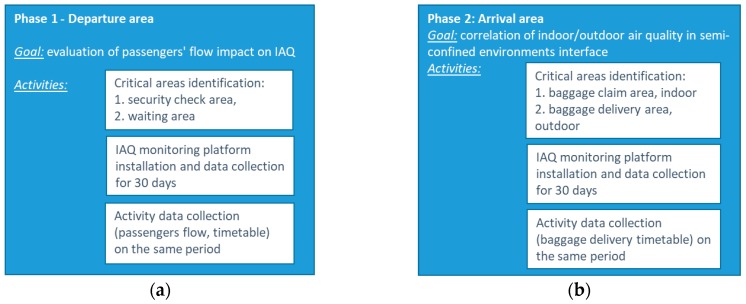
Experimental set-up and flow chart of activities, for phase 1 (**a**), when Departure area of the Terminal was involved, and phase 2 (**b**), when Arrival area was investigated.

The experimental set-up has been organized in two different monitoring periods, during summertime, within two different areas of the terminal, and developed as follows (Figure 3).

### 2.1. Phase 1

Two monitoring platforms have been placed into two different areas within the departure zone of the terminal for a period of 30 days. In particular, security check and waiting for boarding area have been investigated. IAQ data collected have been treated statistically, identifying possible singularity and repetitive patterns in the typical day of standard airport activity. For this purpose, instant concentration data, collected every 5 min by the monitoring platform, have been aggregated into hourly average values. 

In order to evaluate the reliability of data registered, considering the peculiarity of the monitoring technology applied, results obtained have been compared with data collected into three indoor environments (i.e., households) located in the same urban area as the airport terminal, acting as control group. In particular, hourly average values have been compared with data collected into control environments. 

Micro-climatic parameters (i.e., temperature and humidity) have been tested during the whole period and treated statistically in accordance with airborne contaminants concentrations. Additional information has been gathered on each area’s occupation, with particular regard to passengers’ flow data, organized on hourly basis, as to be compared with contaminants trends outlined.

### 2.2. Phase 2

Following the Phase 1 experimental set-up, two monitoring platforms has been installed for a period of 30 days on each side of a baggage claim belt, landside and airside, into the arrivals area of the terminal. IAQ data collected have been treated statistically, identifying possible singularity and repetitive patterns in the typical day of standard airport activity. Particular attention has been paid to possible correlation between indoor and outdoor air quality values, due to the connection between the two environments, represented by the portcullis for baggage delivery. For this purpose, two key parameters (VOC and particulate matter) have been evaluated as indoor and outdoor profiles over the typical day. Additional information has been gathered on each area’s occupation, with particular regard to baggage delivery registration and consequent passengers’ flows, organized on an hourly basis, as to be compared with contaminants trends outlined. Data collected by CO and NO_2_ sensors, equipped on a single monitoring platform, has been evaluated over the two testing periods, in order to compare contaminants profile over the typical day in indoor and outdoor conditions.

An additional step of the study is carried on temperature and humidity in order to investigate passenger’s thermal well-being. At first, a validation step for data collected has been performed, comparing dataset measured on landside terminal areas and the nearest Environmental Authority (Agenzia Regionale per l’Ambiente, Emilia-Romagna Region—ARPAE) long-term monitoring station. In particular, in order to overcome possible singularity and specific events, a statistical treatment of registered data, based on hourly average values, has been applied.

Once verified the reliability of data trends and set reference accordingly with ARPAE records, data have been evaluated in comparison with national standards on energy efficiency and environmental comfort. In particular, indoor environments treated with heating, cooling and venting systems (HVAC) are recommended to be conditioned, during summertime, i.e., the experimental monitoring period for the present study, to temperatures above 26 ± 2 °C and relative humidity ranging between 50% and 60%, following regional regulation, as well as national and international guidelines [30,31,32,33].

## 3. Results and Discussion

Results gathered and elaborated as presented in the previous section are reported in the following, accordingly with the stepwise experimental set-up. 

In particular, the first round of monitoring (Phase 1) is reported in terms of environmental conditions (T and RH%) and airborne pollutants (VOC, odorous gases, PM_1_, PM_2.5_, and CO_2_ equivalent), with average weekly values and variation indexes (SD and SE) in both areas investigated (i.e., security check area and waiting for boarding area). Parameters detected have been, then, compared with passengers’ flow, in order to assess the influence of activity intensity in the area over IAQ. A statistical analysis of average values registered on daily basis has been performed to highlight fluctuations within the week. Finally, data collected have been compared with a set of households monitored in the same period and same urban area, as to provide a benchmark of exposure to airborne pollutants. The very same analysis has been performed on concentration and environmental data collected during the second phase (Section 3.2. Phase 2), during which the focus was set to baggage claim area, both landside and airside. In this case, a direct comparison between the two areas was carried out. 

The study was completed with a micro-climatic assessment (Section 3.3. Microclimatic Evaluation), in terms of temperature and relative humidity.

### 3.1. Phase 1

The first validation step has been successfully concluded, with evidence of the general correspondence of data collected into airport environments with airborne contamination parameters detected into a three-household control group.

As security check area is characterized by a steady presence of staff, passengers’ flow is related to daily flights schedule. Contaminants’ trends have been registered and elaborated on an hourly basis. Table 1 reports the average weekly values and Figure 2 reports hourly average values recorded on daily basis for airborne contaminants, in relative terms of percentage ratio over the minimum value registered (typically correspondent to 3 a.m. value). VOCs and odorous gases sensors registered quite similar trends, while CO_2_eq follows early morning trend (from 5 a.m. to 9 a.m., approximately), displaying secondary peaks on 2 p.m. and 7 p.m., with lower variation compared with the minimum. Particulate matter sensors appear to register lower variations in the daily trends.

**Figure 4:**
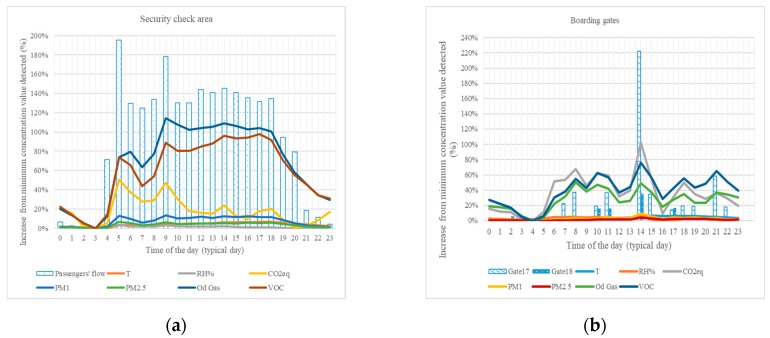
Correlation between level of activity (i.e., passengers’ flow) and airborne contaminants in terms of variation from the minimum value: (**a**) security check area; and (**b**) waiting for boarding area.

Passengers’ flows at security checks are reported as columns in Figure 4a. As evident from the overlapping and similarities between passengers’ and airborne contaminants data, the level of activity in the area seems to be reasonably imputed for the variation in IAQ. In particular, peaks registered in the number of passengers could be regarded as trigger for higher gases concentrations, initially recovered by the air treatment system and then, during the day, cumulating in the environment. As evident from the comparison between Figure 4a,b, the activity rate of the waiting for boarding area is quite discontinuous, as the specific boarding gates are involved only in medium-long range flights, with recurring crowding around 2 p.m. (Figure 4b).

Passengers’ flows resulted the main driving force in gaseous contaminants’ concentration trends (i.e., VOC, odorous gases (OD), and CO_2_ equivalent), displaying similar behavior during the typical day. The primary peak in the area’s activity, registered again, at 2 p.m., in this case outnumber the peak registered on airborne contaminants (e.g., 225% increase over the minimum registered value in passengers’ flow against 100% increase over the minimum registered value in VOC). Particulate matter, on the other hand, shows lower variability on the typical day profile (i.e., values range in a +8% range over the minimum).

In the following Table 2 and Table 3, a statistical evaluation of values monitored on the different days of the week has been performed in comparison with average values obtained on the standard week, in the security check area. The data show remarkable fluctuations; a *t*-test has been performed to evaluate if the differences between average daily values of concentration could have been generated by similar processes or arise from different determinants. The computed *p*-values highlights day-by-day significant differences due to environmental and/or external factors, in particular for PM_10_ (five days out of seven) which seems to be more closely related to the number of passengers present than the other pollutants considered (see Figure 4a).

Contamination patterns have been evaluated over the different days of the week, in terms of hourly averages, in order to spot possible significant difference between each day and the reference average, i.e., “standard week”, in the security check area.

**Figure 5:**
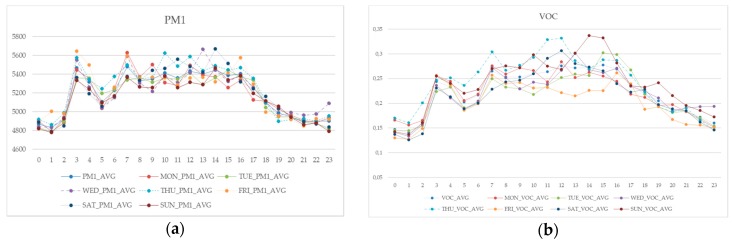
Hourly average values for particulate matter (**a**) and volatile organic compound (**b**) over different days of the week.

As evident from Figure 5, trends appear remarkably similar, with higher differences around lunch time, when the weekend’s days show peaks, both for PM_1_ and VOC. Even in summer flight schedule, typically organized over vacations in fact, weekends are generally characterized by peculiar routines.

Considering the relative basis of values measured by sensors equipped, a reference set of values has been established by monitoring IAQ into three different households located in the same urban area as the terminal airport. Hourly average values have been calculated and a range of acceptability has been identified consequently. The following Table 4 reports interval limit values for each parameter monitored, together with average values calculated over the standard week. As evident from the values reported, terminal airport IAQ and micro-climatic parameters appear in line with household’s environment Even with a limited benchmark of households monitored and considering the intrinsic differences between airport terminal and household environment, in terms of ventilation and occupation, it is reasonable to assess that passengers’ exposure to airborne contaminants tested is basically similar in the two environments.

### 3.2. Phase 2

**Figure 6:**
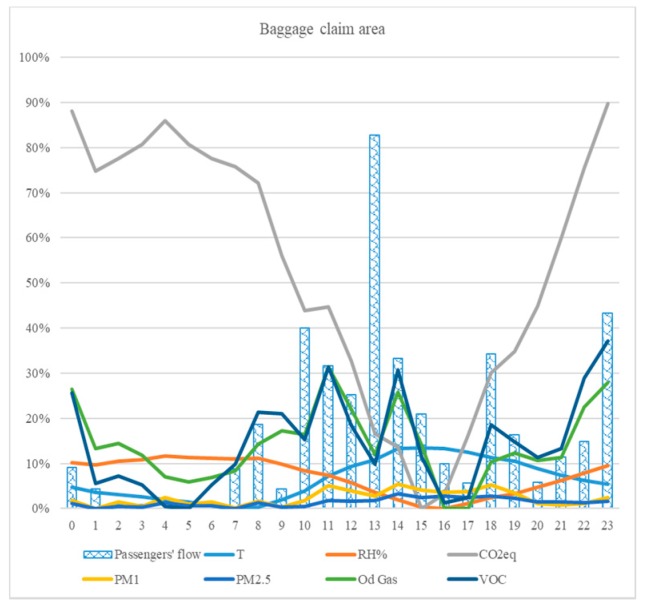
Correlation between the level of activity (i.e., passengers’ flow) and airborne contaminants in terms of variation from the minimum value, baggage claim area.

Airborne contaminants concentrations registered as hourly average on the typical day are reported in Table 5 and Figure 6, together with flights’ schedule and expected passengers’ flow in the area, treated accordingly. 

VOC and odorous gases concentrations detected appear directly related to the level of activity in the area, supporting Phase 1 observations. CO_2_ equivalent, on the other hand, registers a peculiar trend, with minimum values correspondent with central hours of the typical day and maximum values at nighttime. This suggesting a direct correlation between CO_2_ equivalent concentration and area’s specific activity (i.e., baggage delivery), where portcullis opening for baggage delivery and consequent natural ventilation from outdoor interface appears to affect this parameter remarkably. PM trends appear scarcely affected by working conditions of the area, thus confirming Phase 1 results.

**Figure 7:**
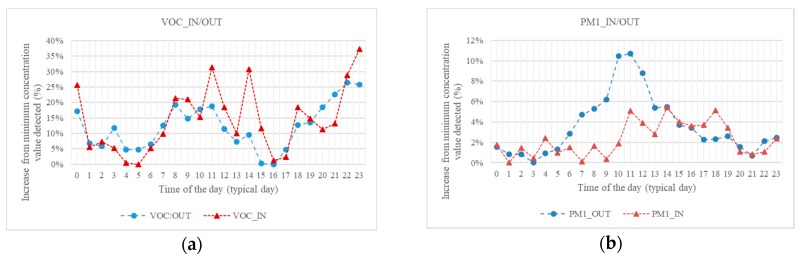
Concentrations in baggage delivery area, indoor (IN) and outdoor (OUT) in terms of increase from the minimum value registered, typical day: (**a**) VOC; and (**b**) particulate matter.

A comparison of airborne contaminants profile, detected at the interface outdoor/indoor, has been performed and the results obtained show, for VOC, an evident correlation between the two environments, with minimum values in the small hours and afternoon and peaks in the central hours of the typical day and at nighttime (possibly following what detected on CO_2_ equivalent sensor), but with higher variation over indoor values. This suggests that the indoor environment, in this case, is both affected by the anthropic activity indoor and outdoor (Figure 7a). 

For particulate matter, on the other hand, a limited range of variation is identified both indoor and outdoor (Figure 7b). Nevertheless, the different shape of the curves suggests that, in the indoor environment, this class of contaminants is reasonably under control, regardless of the level of activity in the area.

### 3.3. Microclimatic Evaluation

The initial validation process applied over temperature and humidity sensors returned different results, depending on the parameter tested.

In particular, temperature sensor presented a systematic error of 2.6 °C, easily set in the post-processing phase, but a quite reliable detection of temperature variations. Therefore, considerations based on temperature detection could be reasonably regarded as reliable, even with the limited accuracy reported by manufacturer (i.e., ±2 °C), considering the scope of the present study.

On the other hand, relative humidity registered by monitoring platform implemented, returned flattened values for the upper range, compared with ARPAE results. It was not possible to discern whether differences are to be attributed to the different location of the two monitoring platforms, with consequences on local occurrences, or to lower sensibility of the implemented sensors in the upper range, at present stage of the study. Therefore, humidity data have been evaluated considering the limitation emerged.

**Figure 8:**
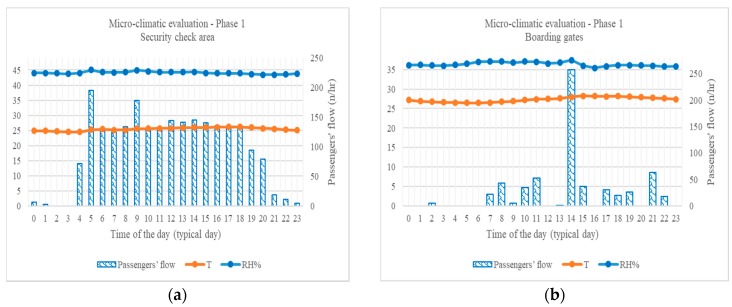
Micro-climatic evaluation, Phase 1, in terms of temperature and relative humidity, typical day profile: (**a**) security check area; and (**b**) boarding gate area.

The security check area (Figure 8a) shows a temperature trend comprised in the two grades tolerance range around 26 °C defined by the reference regulation, thus testifying the HVAC efficiency on this issue. Different scenario is registered at the boarding gate area (Figure 8b) in which no temperature below 24 °C (i.e., reference value) was registered: its values are much higher than ones registered into the security check area, due to:

- direct solar irradiation by panoramic glass wall facing airside of the terminal;
- typical difficulty in ventilating open spaces uniformly; and
- heat generated by passengers waiting in the area for long periods: the average energetic metabolism by sedentary human activities is typically estimated in the range 105–180 watts [25].

**Figure 9:**
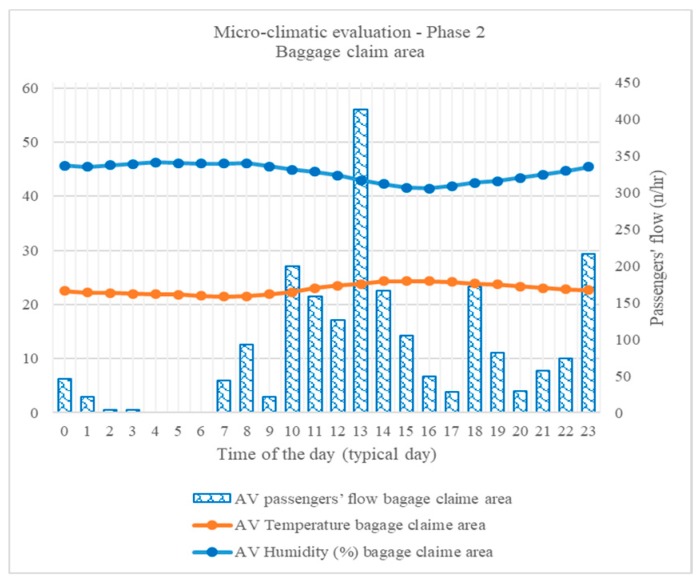
Micro-climatic evaluation, Phase 2, baggage claim area, in terms of temperature and relative humidity, typical day profile.

The differences in structure and use of area investigated during Phase 2 are reflected in the temperature and relative humidity values registered. Temperature hourly average values results compliant with the reference only during afternoon (i.e., 1 p.m. to 7 p.m.), while the rest of the typical day profile is permanently lower (Figure 9). Relative humidity profile appears varying in a wider range than ones registered in Departures areas and permanently below the reference range.

A correct interpretation of such micro-climatic conditions profile required a focus on outdoor data registered during the same period, due to direct indoor/outdoor interface characterizing the area. A sudden change in meteorological conditions was registered by the outdoor monitoring platform during the second monitoring period, thus evidently affecting micro-climatic conditions indoor, even considering methodological limitations [34].

## 4. Conclusions

Since the scope of the study was set in a preliminary characterization of IAQ into key areas of the terminal of a medium-sized airport, four areas have been selected as representative of possible criticalities in IAQ management. A WSN technology has been installed for a period of 30 days to evaluate a set of airborne contaminants, as well as micro-climatic parameters (i.e., temperature and humidity). IAQ data collected have been validated through comparison with references provided by a three-household control group characterized during the same period, placed in the same region. Micro-climatic parameters have been compared with a stable monitoring station powered by ARPAE in the proximity of the airport, finding a good correlation between the two temperature profiles, while humidity values displayed remarkable differences, especially in the upper range.

Phase 1 was aimed to assess the impact of passengers’ flow over IAQ in specific areas of Departure zone of the terminal. Results obtained allowed to identify an evident element of solicitation represented by the typical activity rate (i.e., passengers’ flow through security checks and waiting at the boarding gate) within the areas investigated for gaseous contaminants (i.e., VOC, CO_2_ equivalent, and odorous gases), while particulate matter presents a narrow range of variation of the typical day profile, with only limited dependence on areas occupation.

Phase 2 returned results in line with Phase 1, confirming the correlation between gaseous contaminants trends and the level of activity in the area, even at the outdoor interface (i.e., on the airside of baggage delivery belt). Nevertheless, a peculiar profile has been detected on CO_2_ equivalent sensor, partially coupled with VOC ones, showing an accumulation of gases in the indoor environment during nighttime and low operational periods (i.e., when indoor/outdoor interface is most likely closed). This would suggest the necessity of improving the efficiency of the air ventilation/treatment system to support the removal activity on the gaseous contaminants. At the same time, particulate matter counts result scarcely variated over the typical day profile, both with regards to the location of the sensor (indoor/outdoor, with almost undetectable variations in indoor) and to the activity level, thus testifying an adequate management of the specific contaminants performed by the actual HVAC system.

HVAC system’s performance appears, therefore, efficient in terms of *filtration*, following CDC-NIOSH definition [35], but it seems not able to provide an *air cleaning* (i.e., gases and vapors removal) sufficient to respond to solicitation deriving from anthropic activity in the area. Areas investigated, in fact, are characterized not only by passengers’ flow, but, in the case of the departures area, by commercial and food-preparing activities, such as coffee bars and restaurants. As widely documented [36], food preparation may be regarded as a major source of airborne contamination in indoor (not industrial) environments, in particular of VOCs and particulate matter [35,36,37,38,39]. A closer control on such emissions could lead to a better IAQ management in the departure area. CO and NO_2_ trends followed the expected complementary profile, related to temperature and solar radiation cycles during the day. The possibility of discerning the airside activity contribution from background traffic, considering the highly populated area where the case study airport is set, is left for future study developments. The evaluation of micro-climatic comfort within the investigated areas returned differentiated conditions: while security check area, in fact, presented quite consistent climatic conditions, waiting for boarding area, as well as baggage delivery area, appeared not completely compliant with reference temperature and, particularly, humidity range. These results should be evaluated considering several issues:

1. Reliability of results: discrepancy detected on humidity sensor readings compared with the standard ARPAE monitoring platform, suggests that further investigations would be required in order to draw definite conclusions on this point; and
2. Expected exposure rates for staff and travelers: The arrivals area (in our case, the baggage claim area) is generally characterized by shorter permanence of travelers and discontinuous activity performed by the staff, therefore the perception of micro-climatic comfort is limited, while a closer control over the same parameters into departure area would lead to more significant improvement in travelers’ experience and staff routine. In this sense, the present management over security check area appears satisfying.

On the basis of results obtained from this preliminary characterization phase, it is possible to outline the great opportunity offered by a real-time monitoring network for IAQ and micro-climatic comfort parameters in airport environment in the perspective both of the sustainable management of the facility and travelers experience improvement.

## Figures and Tables

**Table 1:** Average weekly values for T, RH%, CO_2_, PM_10_, PM_2.5_, OD gases, and VOC (Phase 1—departures area).

*Security Check Area*	T (°C)	RH%	CO_2_eq	PM_1_	PM_2.5_	OD GAS	VOC
Avg.	25	44.09	1776.92	5196.33	1789.44	0.8168	0.2238
SE	0.0089	0.0448	13.7	4.96	0.9301	0.0026	0.0009
SD	0.8172	4.09	1253.13	454.41	85.04	0.2338	0.0798
min	23	30	450	4240.85	1538.23	0.3398	0.0625
max	28	51	7370	12,156.16	2642.16	1.9766	0.8281
***Wait Boarding Area***							
Avg.	27	36.38	1230.43	5467.78	1995.7	1.629	0.051
SE	0.013	0.038	6.535	5.213	1.46	0.005	0
SD	1.169	3.5	597.37	476.52	133.46	0.477	0.023
min	25	25	450	4226.45	1606.56	0.875	0.011
max	31	47	5873.66	12,991.38	3844.96	15.45	0.546

**Table 2:** Statistical evaluation of average PM_10_ and VOC values obtained on the different days of the week and average values of the standard week in security check area (average value, standard deviation, and *t*-test *p*-value).

	PM_10_			VOC		
	Avg.	Std. Dev.	*p*-Value	Avg.	Std. Dev	*p*-Value
**Standard week**	5196.33	454.42		0.2238	0.0798	
**MON**	5189.73	407.80	0.6195	0.2227	0.0808	0.6614
**TUE**	5171.39	417.98	0.0769	0.2164	0.0819	0.0033 *
**WED**	5208.13	440.05	0.3986	0.2209	0.0671	0.2391
**THU**	5254.04	470.11	0.0001 *	0.2423	0.0710	0.0001 *
**FRI**	5216.93	488.38	0.1649	0.2035	0.0659	0.0001 *
**SAT**	5200.46	473.79	0.7803	0.2162	0.0702	0.0021 *
**SUN**	5146.13	466.15	0.0005 *	0.2403	0.1016	0.0001 *

(*) Sign. for *p* < 0.05.

**Table 3:** Statistical evaluation of average PM_2.5_ and OD values obtained on the different days of the week and average values of the standard week in security check area (average value, standard deviation and *t*-test *p*-value).

	PM_2.5_			OD		
	Avg.	Std. Dev.	*p*-Value	Avg.	Std. Dev	*p*-Value
**Standard week**	1789.44	85.04		0.8168	0.2338	
**MON**	1796.03	79.84	0.013 *	0.8168	0.2184	0.9999
**TUE**	1792.77	78.02	0.2073	0.8009	0.2350	0.0306 *
**WED**	1791.42	81.71	0.4554	0.8019	0.2313	0.0423 *
**THU**	1801.16	87.08	0.0001 *	0.8341	0.2250	0.0180 *
**FRI**	1782.87	88.22	0.0145 *	0.7845	0.2106	0.0001 *
**SAT**	1785.25	91.26	0.1203	0.8161	0.2194	0.9236
**SUN**	1779.16	85.99	0.0001 *	0.8542	0.2737	0.0001 *

(*) Sign. for *p* < 0.05.

**Table 4:** Results obtained on the standard week (average values) compared with control environments.

	T (°C)	RH%	CO_2_eq	PM_1_	PM_2.5_	OD GAS	VOC
**Avg_MIN_House**	21	29.72	471.75	4199.10	1458.95	0.7030	0.147
**Avg_MAX_House**	31	45.80	1518.72	7607.98	2065.18	3.008	1.403
**Standard week**	25	44.09	1776.92	5196.33	1789.44	0.816	0.223

**Table 5:** Average weekly values for T, W, CO_2_, PM_10_, PM_2.5_, OD gases, and VOC (Phase 2—arrivals area).

*Bag. Claim Landside*	T (°C)	RH%	CO_2_eq	PM_1_	PM_2.5_	OD GAS	VOC
Avg	23	44.37	3394.21	5594.75	1872.38	0.3971	0.1091
SE	0.0150	0.051	20.75	5.341	0.9860	0.0011	0.0004
SD	1.654	5.608	2280.85	587.03	108.37	0.1220	0.0405
min	15	30	0	4641.89	1659.90	0.0977	0.0391
max	29	63	11,329.33	27,540.84	4066.01	2.2305	0.7344
***Bag. Claim Airside***							
Avg	22	44.68	5526.16	7377.03	2579.10	0.42	0.022
SE	0.037	0.083	31.39	7.97	1.0056	0.0023	0.0001
SD	4.114	9.207	3465.24	879.81	111.01	0.2549	0.0095
min	11	21	450	5828.05	2228.17	0.2461	0.0039
max	36	67	16,391.66	18,107.58	3871.45	16.48	0.2305
